# miR-494 induces EndMT and promotes the development of HCC (Hepatocellular Carcinoma) by targeting SIRT3/TGF-β/SMAD signaling pathway

**DOI:** 10.1038/s41598-019-43731-4

**Published:** 2019-05-10

**Authors:** Jinqian Zhang, Yan Zhu, Liangshan Hu, Fang Yan, Jinglong Chen

**Affiliations:** 1 Department of Laboratory Medicine and Central Laboratories, Guangdong Second Provincial General Hospital, Guangzhou, 510317 P.R. China; 2 Department of Medical Experiment Center, Guangdong Second Provincial General Hospital, No. 466 Xingang Middle Road, Haizhu District, Guangzhou, 510317 Guangdong Province China; 3 Department of Oncology, Beijing Ditan Hospital, Capital Medical University, Beijing, 100015 P.R. China

**Keywords:** Cancer therapy, Tumour angiogenesis

## Abstract

EndMT has an important effect on metastasis and progression of tumor. This work will elucidate the effect of miR-494 on EndMT and development of HCC. Therefore, the differential miRNA expression among non-tumorous, para-tumorous and tumorous tissues was analyzed. Moreover, luciferase activities of SIRT3 3′UTR treated with miR-494 were determined. Then human hepatoma cell lines were dealt with mimics or inhibitors of miR-494, migration and proliferation ability were assessed. The expression of SIRT3 and markers of mesenchymal cell were analyzed. The influences of miR-494 on development of HCC through inducing EndMT by targeting SIRT3 and TGF-β/SMAD signaling pathways in hepatoma cell lines were investigated. Xenograft mice were used to explore the potential roles of miR-494 on EndMT and development of HCC *in vivo*. Our results showed that, compared with non-tumorous tissues, 17 miRNAs were upregulated and 3 miRNAs were down-regulated in tumor tissues. In tumor tissues, the miR-494 expression level was much more than the expression of para-tumorous and non-tumorous tissues. MiR-494 suppressed SIRT3 expression, additionally enhanced expression of mesenchymal cell markers, while exerted effects on cell proliferation and migration of hepatoma cell lines. Moreover, the antagomir of miR-494 could protect against development process in xenogarft murine model. In conclusions, our work demonstrated that miR-494 targeted to SIRT3, and was a crucial mediator of EndMT and development of HCC through regulating SIRT3/TGF-β/SMAD signaling pathway. It suggested that aim at SIRT3/TGF-β/SMAD signaling pathway through suppressing the miR-494 expression level, was a feasible therapy strategy for HCC.

## Introduction

HCC (hepatocellular carcinoma) is predominantly constitutive of primary liver cancer. Moreover, the morbidity of HCC is the fifth most among all the cancers, and its mortality rate is third in the world, just subsequent to lung cancer and gastric cancer^1–3^. Due to its difficult early diagnosis, easy early metastasis, rapid development and lack of effective therapy, the five-year survival of it was about 5~15%^1,4^. The pathogeny of HCC may be associated with persistent liver damage, such as chronic HBV or HCV infection, exposure to heavy alcohol consumption, smoking or aflatoxin, or may be direct genotoxic substance, all of which are of significant relevance^2^. Most patients sequentially develop hepatitis, fibrosis, cirrhosis, and then HCC. The biggest risk factor related to carcinogenesis of hepatocytes is cirrhosis, which comes from long latencies of chronic hepatic inflammation and injury for 20–40 years^4^. More than 80% HCC developed from fibrosis or cirrhotic livers, it also indicate long-term biological, chemical or physical damage that is playing an important role in hepatocarcinogenesis^5^.

EndMT (Endothelial to mesenchymal transition) is the critical course, and plays an important role in metastasis and progression of tumor. The abilities of invasion and migration increase, and epithelia polarity vanishes away during this process together with up-regulation of mesenchymal markers and down-regulation of epithelial markers^6–13^. Some transcription factors regulate the process of EndMT, including Zeb1, Slug, Snail, TGFβ, and Twist^9,13^. The biological process of EndMT is complex, including losing characteristics of endothelia, and acquiring mesenchymal phenotypes. Moreover, Smad 3, α-SMA (alpha smooth muscle actin), etc, which were the markers of mesenchymal cell, expression of them were induced^14,15^. Tumor growth relies not only on the malignancy of the tumor cells but also on the interaction between tumor cells and stromal cells^14,15^. The system of tumor vessel includes pericytes, smooth muscle cells and, endothelial cells (ECs), which plays and important role in tumors progression and angiogenesis activation^16,17^. EndMT process of ECs is conduced to the progression of cancer, that has been identified by recent researches^18–20^. In this process, the properties of migration or invasion and markers of mesenchyma (e.g., α-SMA, FSP-1) are obtained, the endothelial markers (e.g., CD31, VE-cadherin) of ECs and junctions of cell-cell lose, as well as the phenotype characterization of mesenchyma is acquired^21^. These investigations confirm that all kinds of pathological processes occurs with, for example, cancer^18,19,22^.

Lately, some scholars have revealed that Sirtuin3 (SIRT3) plays a major role in repairing cell injury, inhibiting cell necrosis and cell canceration^23–25^. As one of the seven sirtuin gene families, it shows activity of histone deacetylase, and localizes greatly in mitochondria, and playing vital role in cell development and reparation^26^. For example, SIRT3 is necessary to prevent age-related hearing loss, reduce oxidative stress and mediate damage by caloric restriction^27,28^. The mechanism that SIRT3 attenuates dysfunction of activated cells is closely related to mitochondrial function, which includes to reduce opened mitochondrial permeability transition pore (MPTP) and protein expression of cyclophilin D (CypD), inhibit mitochondrial CYT-C release to cytoplasm, reduce ratio of Bax/Bcl-2 and activity of caspase-3/9^24^. On the contrary, the deficiency of SIRT3 makes cell fail to repair from damage, whereas continuous injury can induce cell carcinogenesis^25,29^. Therefore, SIRT3 can work as a cytoprotective factor and mitochondrial tumor suppressor^29^. Built on the foregoing, the hypothesis that up-regulated expression SIRT3 may be a potential anticancer strategy for tumor therapy. Our previous study found the interaction between NMNAT2 and SIRT3 can regulate cell apoptosis and proliferation process of NSCLC cell lines^30^.

A variety of evidences had confirmed that microRNAs were the crucial mediator in activity of EC and EndMT^31,32^. Therefore, our work studied the relationship between early recurrent HCC and up-expression of miR-494 through comparing miRNA expression profiles from liver tissues of patients with HCC. Over-expression of miR-494 plays the positive feedback role in TGF-β pathway through restraining expression change of SIRT3 and its pathway associated with EndMT in HCC development.

## Materials and Methods

### Specimens of patients

The tissues of patients with HCC were obtained from January 2014 to December 2017. All these patients were performed with hepatectomy, and did not receive any treatment before hospital. At the operation day, samples of tumor tissues, para-tumorous tissues and non-tumorous tissues were collected and handled immediately, respectively.

This investigation and the utilization of human specimens in this work had been approved by the Ethics Committee of our hospitals (Guangdong Second Provincial General Hospital, Guangzhou, China; and Beijing Ditan Hospital, Capital Medical University, Beijing, China) on the basis of the Declaration of Helsinki. We clearly confirmed that the informed consents were obtained from all patients. We had recorded and documented participant consent in our hospital.

### Extraction of miRNAs and next-genersequencing

The miRNA-sequencing test was conducted after extraction of RNAs with TRIzol method and miRNeasy Mini Kit. Furthermore, total RNA was harvested from tissues for RT-qPCR assay with mirVana™ PARIS™ Kit. Hybridization and ligation were conducted using Adaptor Mix. Moreover, microRNA sequencing RNAs using Illumina HiSeq. 2000 platform was conducted after reversely transcribe of these RNAs.

### Data extraction and analysis

Data extraction and analysis was performed as showed in Flow diagram (Supplement Figure 1). All the mature miRNAs sequences were identified accompanied by 5 flanking nucleotides through library preparation of reference sequences with miRBase (version 19.0)^33^. Given that the length of minimum read was 50 nucleotides, specific adapters were used to anneal to their 3′ ends and extended the entire small RNAs during the library preparation. Thus, we removed the adapters and obtained the potential miRNAs with the length of 15–30 nucleotides for further analysis using cut adapt software in silico^34^. Subsequently, the sequences were perfectly matched with the prepared reference library with Bowtie (version 0.12.7)^35^. Then the numbers of mapped reads were computed for every miRNA. Acquired data of all samples were normalized with RPM (Reads per Million)^36^.

### RNA extraction

TRIzol method was used to extract total RNA from tissues based on the operation manual. Then, smaller RNA (<200 nt) was removed with mirVana RNA isolation kit according to the instructions for manufacturer.

### Culture of cell

The cell lines of human HCC, including HepG2 (ATCC^®^ HB-8065^TM^), Hep3B (ATCC^®^ HB-8064^TM^), SNU-449 (ATCC^®^ CRL-2234^TM^), PLC/PRF/5 (ATCC^®^ CRL-8024^TM^), SK-HEP-1 (ATCC^®^ HTB-52^TM^), and PLHC-1 (ATCC^®^ CRL-2406^TM^), were purchased from ATCC (American Type Culture Collection), and then cultured in DMEM with 15% FBS accompanied by Streptomycin and Penicillin (bi-antibiotics) with atmosphere (5% CO_2_) at 37 °C.

### qRT-PCR

The expression level of miR-494 in tissues was assessed with TaqMan miRNA assays. The data analysis was performed with 2^−ΔΔCt^ method. The qRT-PCR assay was conducted using SYBR Green kit. The primer sequences of miR-494 were 5′-ATTGGAACGATACAGAGAAGATT-3′ (upstream) and 5′-GGAACGCTTCACGAATTTG-3′ (downstream), respectively. Moreover, the primer sequences of U6 were 5′-TGACCTGAAACATACACGGGA-3′ (upstream) and 5′-TATCGTTGTACTCCACTCCTTGAC-3′ (downstream), respectively.

The primer sequences of SIRT3 were 5′-AGGTCGGGAGCGTCTTGTAG-3′ (upstream) and 5′-CATGAACCCCTCATCTTCCTGAG-3′ (downstream), respectively. The primer sequences of TGF-β were 5′-CAACACGATGCTTGAAGGTAACG-3′ (upstream) and 5′-TCCAGAGAGATGATTGCCGAGG-3′ (downstream), respectively. Moreover, the primer sequences of GAPDH (glyceraldehyde-3-phosphate dehydrogenase) were 5′-CTCATGACCACAGTCCATGCC-3′ (upstream) and 5′-GGCATGGACTGTGGTCATGAG-3′ (downstream), respectively.

### Reporter gene assays

The online software Targetscan (version 7.2) was firstly used to forecast the microRNA which could regulate the expression of SIRT3. Therefore, miR-494 was acquired from predict result.

Moreover, the 3′UTR mRNAs sequence of SIRT3 was cloned into pmirGLO vector with luciferase reporter firefly and renilla. The control was used with reverse orientation of SIRT3 3′UTRs mRNA. The complementary region sequence in SIRT3 3′UTR was UUUC(AAGAUG)AUGUUUC (Supplementary Table 1), which located at position 1618–1624, and corresponding miR-494 seed sequence CUCCAAAGGGCACAUACAAAGU. Site-Mutation kit was used to constructed SIRT3 3′UTR mutated vector. Finally, Dual-Glo ® Luciferase Assay System was used to analyze the activity of luciferase with analyzer VICTOR.

### Immunohistochemistry assay

Tissue samples were prepared using 10% neutral formalin, then embedded using paraffin after dehydration. Furthermore, it was cut into thick sections with 5 mm, dewaxed and hydrated. After antigen retrieval, incubation with primary antibody for these sections was performed. SIRT3 (AffinitY, AF5135, 1:200) was used as the first antibody. The sections were incubated with a DAB substrate kit (Pierce, USA) after incubation with the secondary antibody. These sections were assessed and photographed under a microscope at 200× (IX71; Olympus, Japan). Per sample was taked for 3–5 shots. The expression of these proteins was detected by measuring the optical density of each photograph using Image-Proplus 6.0 software.

### The mimics of miR-494 and its inhibitors

The mimics of miR-494 and its inhibitors were transfected with Lipofectamine 3000. Moreover, the mimics sequences of miR-494 were 5′-UGAAACAUACACGGGAAACCUC-3′ (sense) and 5′-GGUUUCCCGUGUAUGUUUCAUU-3′ (antisense), respectively^37^. Moreover, mimics control of miR-494 were 5′-UUCUCCGAACGUGUCACGUTT-3′ (sense) and ACGUGACACGUUCGGAGAATT-3′ (antisense). The sequence of miR-494 antagomir was 5′-GAGGUUUCCCGUGUAUGUUUCA-3′. The sequence of NC (negative control) was 5′-ACGUGACACGUUCGGAGAAUU-3′ (sense) and 5′-AAUUCUCCGAACGUGUCACGU-3′ (antisense), while it was not consistent with sequences of human genome.

### Western blot

A lysis buffer (150 mM NaCl, 0.1% SDS, 0.02% NaN_3_, 1% NP-40, and 50 mM pH 8.0 Tris) containing cocktail of inhibitor for protease and phenylmethylsulfonyl fluoride (1 mM) was used to lysate cells. Western blotting assays were performed with Biolad Protean II minigel system. Then, 50 μg protein sample was injected in every gel (12%) well of, and then shifted to PVDF membrane after electrophoresised. 1× TBST (5% dry skimmed milk) was used to dispute the non-specific binding. Then the membranes were sequentially incubated with primary and secondary antibody, respectively. Furthermore, these membranes were determined with enhanced ECL (chemiluminescence) reagent of Hyperfilm ECL kit, and exposed using x-ray film.

The primary antibodies included α-SMA (CST 19245, dilution 1: 1200) and Smad 3 (C67H9) (CST 9523, dilution 1: 1500), p-Smad 3 (CST-9520, dilution 1: 1000), TGF-β (56E4) (CST-3709, dilution 1: 1200), SIRT3 (CST2627, dilution 1: 1000), E-cadherin (24E19) (CST 3195, dilution 1: 800), β-actin (13E5) (CST 4970S, dilution 1: 4000) (Cell Signaling, Danvers, MA, USA).

### Analysis of cell proliferation and migration

The proliferation ability of cells was assessed using MTS kit. Control cells were dealt with DMSO, and experiment cells were exposed to 4βHWE (1, 2, 5 and 10 µg/ml, respectively). After incubation with solution of MTS, cells were determined with Plate Reader at 490 nm. The ability of cells migration was detected using wound scratch assay. After scratching, cells were cultured for another 48 h. Then, the movement of cells was observed. Moreover, migration of cells were numbered.

### Animals

Nude mice (purchased from Laboratory Animal Sciences, Southern Medical University, Guangzhou, Guangdong Province, China) were used to conduct our investigation at this work. They were induced to generate xenograft model, and then housed of Laboratory Animal Center, Southern Medical University, Guangzhou, Guangdong Province, China. Mice were maintained at 20–24 °C (temperature controlled)with 12 h light/dark cycle and standard laboratory diet in a pathogen-free environment. The experimental protocol was approved by the licensing committee of Southern Medical University (Guangzhou, Guangdong Province, China). Moreover, All experiments were performed in accordance with relevant guidelines and regulations. All experiments related to animals were performed based on Laboratory Animal Center, Southern Medical University, Guangzhou, Guangdong Province, China, Ethical Committee Acts.

### Experiment *in vivo*

The experiment was initiated with 6 week-old of mice weighing 20–25 g. HepG_2_ cells were resuspended at the concentration of 1 × 10^6^ cells/ml, 1 ml suspension was injected into subcutaneous tissue of nude mice at right flanks (n = 6/group). While tumor size of mice reached 3–8 mm, the experiment initiated as the 1st day. The antagomir of miR-494 were formulated (1.0 mg/ml in PBS). Sequences are 5′-g_S_a_S_gguuucccguguauguu_S_u_S_c_S_a_S_-Chol-3′ (antagomiR-494) and 5′-g_S_a_S_gguuucccguguuacug_S_g_S_a_S_c_S_-Chol-3′ (antagomiR-494 mutant as a control). The nude mice were divided into experiment and control groups at random, then injected with antagomir of miR-494 (5 µl) or it control once every three days for 6 times and terminated on the 18th day. Then, these mice were sacrificed at the 21th day, and tumor mass was dissected from each mouse. These tumors were cut into two sections, then the volume of tumors were determined every 3 days base on the formulate width^2^ × length × 0.5. Moreover, the expression of SIRT3 and TGF-β were determined with western blotting and immunohistochemical staining assay, respectively.

### Statistics

Welch *t*-test-paired was performed to compare isomiRs deregulation and selection of miRNAs between samples of patients and control. The errors were evaluated with false discovery rate (FDR) due to multiple comparisons. On the basis of the expression profiles, Hierarchical clustering of selected miRNAs was conducted with Ward’s agglomeration. Target Rank software was performed to identify the significantly deregulated each seed sequence of miRNA in target genes between samples of patients and control^38^.

The differences between two groups were analyzed with Student’s *t*-test. The continuous variables with normally distributed were represented as mean ± SD (standard deviation). Kruskal-Wallis ANOVA method was used to analyze abnormally distributed data among groups. SPSS (version 18.0) soft was performed for entire statistical analyses. While *P* < 0.05 indicated statistically significant.

### Ethical standards

#### Human rights statement and informed consent

All procedures followed were in accordance with the ethical standards of the responsible committee on human experimentation (Ethics Committee of Guangdong Second Provincial General Hospital (Guangzhou, China) and Beijing Ditan Hospital, Capital Medical University (Beijing, China)) and with the Helsinki Declaration of 1964 and later versions. Informed consent to be included in the study, or the equivalent, was obtained from all patients.

#### Animal studies

All institutional and national guidelines for the care and use of laboratory animals were followed.

## Results

### Differential miRNA expression profiling

To assess the differential expression profiles of miRNAs between para-tumorous and tumor tissues, sequencing experiments of miRNAs were conducted on the total RNA obtained from samples. Among the results of miRNAs-sequencing, 20 were differentially expressed between tumor and para-tumorous tissues. Compared with para-tumorous tissues, 17 miRNAs were upregulated and 3 miRNAs were down-regulated in patients with HCC (fold-change >2.0 and P-value < 0.01) (Table 1).

**Table 1:** Differentially expressed miRNAs in tumor and tumorous tissues (fold-change >2.0 and P-value < 0.01).

miRNA	Fold-change(tumor/para-tumorous tissues)	*t* value	*P* value
miR-146a	5.73	8.381	7.72E-04
miR-221	5.68	9.128	2.07E-03
miR-130a	4.08	6.935	9.33E-03
miR-324-5p	4.01	7.220	7.00E-03
miR-148b	3.91	9.272	5.46E-03
miR-494	3.18	2.702	6.96E-03
miR-23a	2.64	7.254	1.77E-03
miR-30b	2.45	3.823	5.44E-03
miR-301a	2.36	6.256	7.68E-03
miR-223	2.34	3.293	4.42E-03
miR-33a	2.33	5.502	6.51E-03
miR-30c	2.32	7.304	3.81E-03
miR-148a	2.29	2.221	7.31E-03
miR-744	2.27	3.186	8.45E-03
miR-424	2.24	4.571	9.77E-04
miR-17	2.22	5.168	3.26E-03
miR-128	2.08	3.945	3.82E-03
miR-939	0.49	−4.262	6.52E-03
miR-1915	0.35	−0.391	5.49E-03
miR-638	0.34	−0.326	7.53E-03

**Figure 1:**
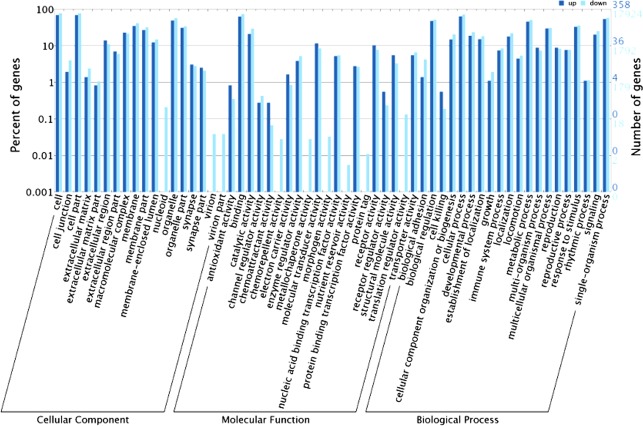
The gene ontology (GO) consortium and data. The gene ontology (GO) consortium and data was used to analyze the targets gene of different microRNAs. The Figure indicated that there was significant expression difference of genes in signaling pathway related to the sequencing microRNAs between tumor and para-tumorous tissues.

The gene ontology (GO) consortium and data was used to analyze the targets gene of different microRNAs. The Figure indicated that there was significant expression difference of genes in signaling pathway related to the sequencing microRNAs between tumor and para-tumorous tissues (Fig. 1).

### The expression level of miR-494 in non-tumorous, para-tumorous, and tumorous tissues

**Figure 2:**
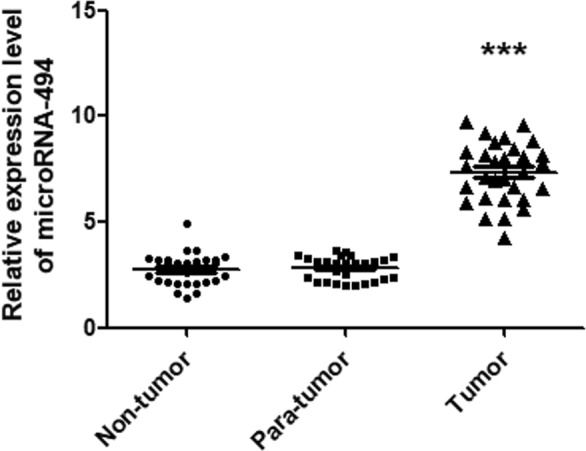
The miR-494 expression level in tumorous, para-tumorous and non-tumorous tissues. The relative miR-494 expression levels in tumor, para-tumorous and non-tumorous tissues of patients (n = 30) were determined using qRT-PCR method. Our work identified the relative miR-494 expression level was obviously much more than that of para-tumorous and non-tumorous tissues. However, there was no obvious difference between para-tumorous and non-tumorous tissues. The data are presented as means ± SD from at least three independent experiments. ***P < 0.001.

Furthermore, the relative miR-494 expression levels in non-tumorous, para-tumorous, and tumor tissues (n = 30) were determined using qRT-PCR method. Our work identified the relative miR-494 expression level was obviously much more than that of non-tumorous or para-tumorous tissues. However, there was no obvious difference between para-tumorous and non-tumorous tissues (Fig. 2).

### Expression levels of miR-494 were determined in cell lines of human HCC

**Figure 3:**
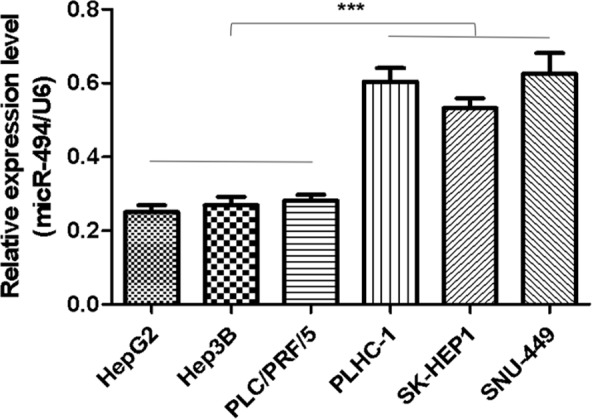
Expression levels of miR-494 were determined in cell lines of human HCC. The relative miR-494 expression levels were investigated in six cell lines of human HCC. Compared with basal epithelial phenotype cell lines PLC/PRF/5, Hep3B, and HepG2, the relative miR-494 expression levels were significantly higher in mesenchymal phenotypic cell lines SNU-449, SK-HEP1, and PLHC-1. The data are presented as means ± SD from at least three independent experiments. ***P < 0.001.

Moreover, the relative miR-494 expression levels were investigated in six cell lines of human HCC. Compared with basal epithelial phenotype cell lines PLC/PRF/5, Hep3B, and HepG2, the relative miR-494 expression levels were significantly higher in mesenchymal phenotypic cell lines SNU-449, SK-HEP1, and PLHC-1 (*P* < 0.001) (Fig. 3).

### MiR-494 inhibited SIRT3 expression in HepG2

**Figure 4:**
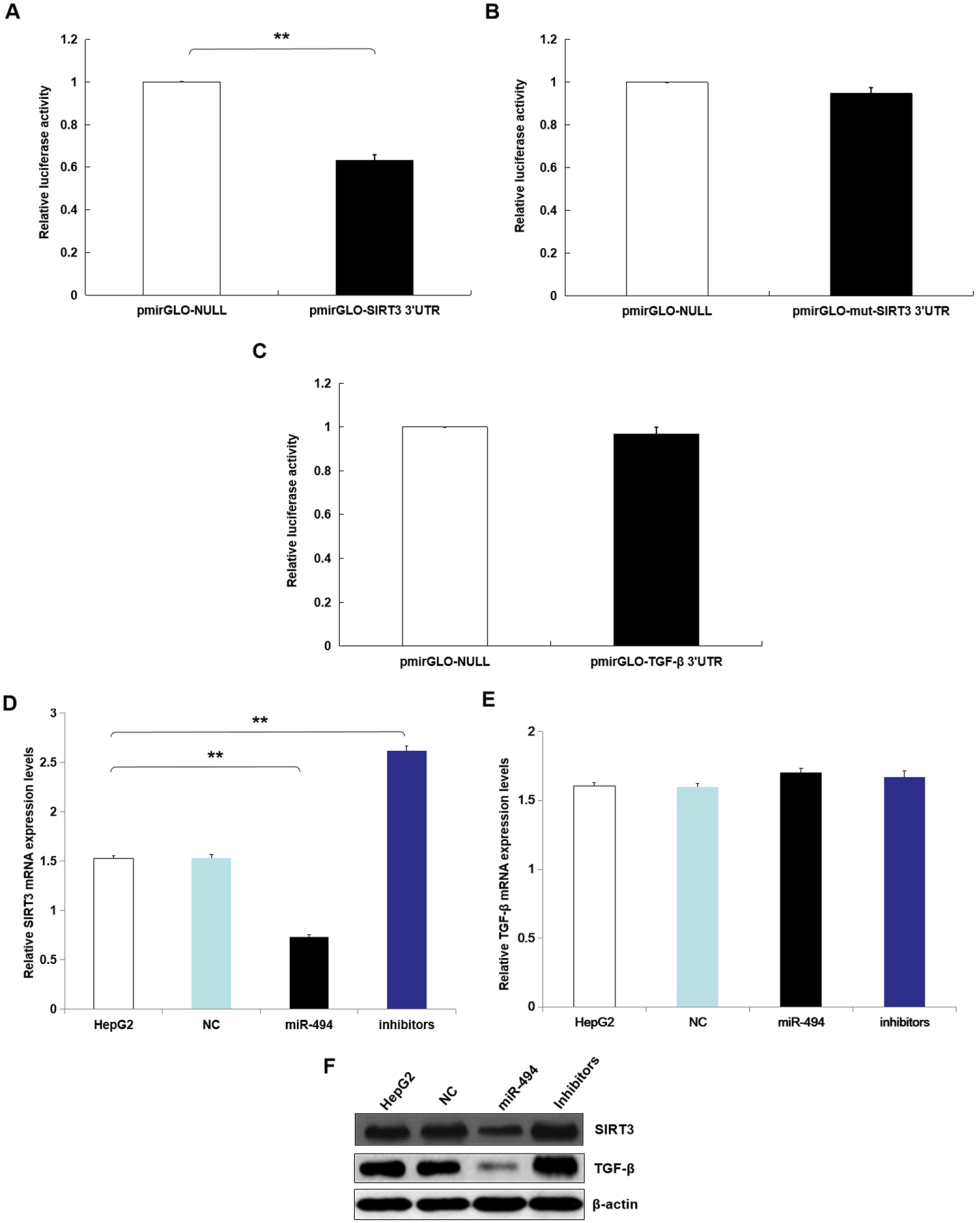
MiR-494 inhibited SIRT3 expression in HepG2. Our data indicated 3′UTR luciferase activities of SIRT3 significantly down-regulated in HepG2 dealt with miR-494. (**A**) However, it did not show obviously change in the group of SIRT3 mutation. (**B**) Moreover, miR-494 did not bind straight to 3′UTRs of TGF-β (**C**), respectively. These data identified that miR-494 targeted to SIRT3 mRNAs. For explore the roles of miR-494 on mRNAs and proteins expression of TGF-β and SIRT3 in HepG2, the qRT-PCR assay and western blotting trial were performed. These results demonstrated miR-494 suppress the expression of SIRT3 mRNAs and proteins in HepG2. (**D**,**F**) However, miR-494 could not suppress the expression of TGF-β mRNAs (**E**), but their proteins expression. (**F**) Moreover, miR-494 inhibitors promoted SIRT3 mRNA expression in HepG2. (**D**) However, miR-494 could not suppress the expression of TGF-β mRNAs. (**E**) The inhibitors of miR-494 could suppress the SIRT3 and TGF-β protein expression of levels. (**F**) The data are presented as means ± SD from three independent experiments. *P < 0.05, **P < 0.01.

At first, the target of miR-494 were predicted with Targetscan, an online software. Therefore, miR-494 was found that possibly aimed to 3′UTRs (untranslated regions) of SIRT3 mRNA (Supplement Table 1). Further experiments were conducted using luciferase reporter gene system to identify whether miR-494 could straight bind with 3′UTRs of SIRT3 mRNA. The data indicated 3′UTR luciferase activities of SIRT3 significantly down-regulated in HepG2 dealt with miR-494 (Fig. 4A). However, it did not show obviously change in the group of SIRT3 mutation (Fig. 4B). Moreover, miR-494 did not bind straight to 3′UTRs of TGF-β (Fig. 4C). These data identified that miR-494 targeted to SIRT3 mRNAs.

To explore the roles of miR-494 on mRNAs and proteins expression levels of TGF-β and SIRT3 in HepG2, the qRT-PCR assay and western blotting trial were performed. These results demonstrated miR-494 restrained SIRT3 mRNAs and proteins expression levels in HepG2 (Fig. 4D,F). However, miR-494 could not suppress the expression of TGF-β mRNAs (Fig. 4E), but its expression level of protein (Fig. 4F). Moreover, miR-494 inhibitors could promote expression of SIRT3 mRNA in HepG2 (Fig. 4D), but could not influence mRNAs expression level of TGF-β (Fig. 4E). Moreover, miR-494 inhibitors could suppress the SIRT3 and TGF-β protein expression levels, respectively (Fig. 4F). In a word, by binding with SIRT3 3′-UTR, the result indicated miR-494 could straight regulate its mRNA and protein expression, further affect protein expression of down-stream genes TGF-β in SIRT3 pathway. It was same as the results of that in other hepatoma cell lines (data not shown).

### SIRT3 expression in non-tumorous, para-tumorous, and tumorous tissues

**Figure 5:**
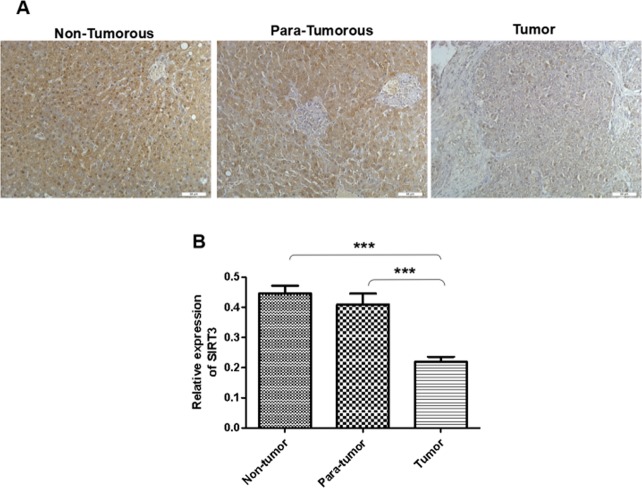
SIRT3 expression in non-tumorous, para-tumorous, and tumorous tissues. The immunohistochemistry results of liver tissues showed that SIRT3 was positive in all cases. However, the positive intensity and express location in different groups were differences. As showed in this Figure, SIRT3 mainly appeared in the cytoplasm in tumor tissue, but they were at the same time appearing in the cytoplasm and nucleus of para-tumorous and non-tumorous tissues. Moreover, the positive intensity of tumor tissue much lower than para-tumorous or non-tumorous tissues. The differences of SIRT3 positive intensity between tumorous and para-tumorous or non-tumorous tissue were statistically significant. The data are presented as means ± SD from three independent experiments. ***P < 0.001.

The immunohistochemistry results of liver tissues showed that SIRT3 was positive in all cases. However, the positive intensity and express location in different groups were differences. As showed in Fig. 5, SIRT3 mainly appeared in the cytoplasm in tumor tissue, but they were at the same time appearing in the cytoplasm and nucleus of para-tumorous and non-tumorous tissues. Moreover, the positive intensity of tumor tissue much lower than para-tumorous or non-tumorous tissues. The differences of SIRT3 positive intensity between tumorous and para-tumorous or non-tumorous tissue were statistically significant (***P < 0.001). In summary, our result indicated the definite relationship between the expression level of SIRT3 protein and its contribution to the occurrence of HCC.

### MiR-494 promoted proliferation or migration of hepatoma cells

**Figure 6:**
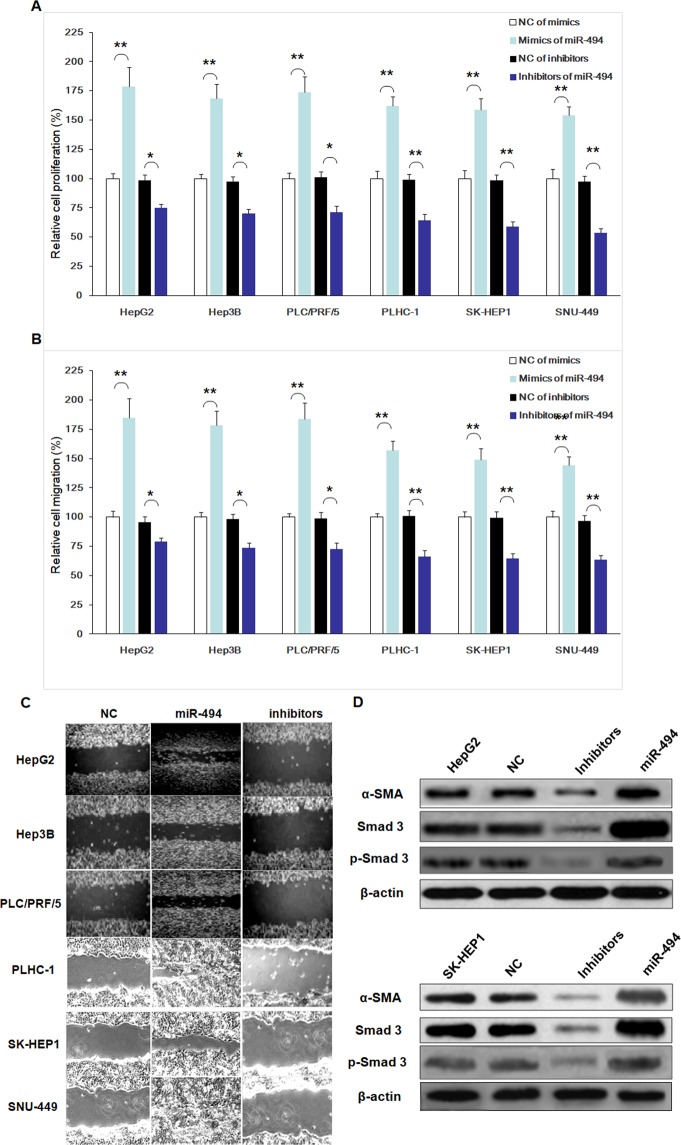
MiR-494 promoted migration or proliferation process of hepatoma cell lines, and induced mesenchymal markers expression of hepatoma cell lines. To assess the biofunctions of miR-494, over-expressed and down-expressed miR-494 was conducted, respectively. Furthermore, several molecular functional tests were performed in hepatoma cell lines. The proliferation ability of hepatoma cell lines was enhanced by miR-494 compared to that of control. (**A**) Moreover, the change of proliferation ability induced by mimics of miR-494 in mesenchymal phenotypic cell lines SNU-449, SK-HEP1, and PLHC-1 was significantly more than that of basal epithelial phenotype cell lines PLC/PRF/5, Hep3B, and HepG2, but the difference was not statistically significant (*P* > 0.05). (**A**) Additionally, the miR-494 inhibitors suppressed proliferation of hepatoma cell lines. (**A**) Furthermore, the change of proliferation ability induced by inhibitors of miR-494 in mesenchymal phenotypic cell lines SNU-449, SK-HEP1, and PLHC-1 was less than that of basal epithelial phenotype cell lines PLC/PRF/5, Hep3B, and HepG2, while the difference between the two type panel of cell lines was statistically significant (*P* < 0.01). (**A**) The migration ability of hepatoma cell lines was promoted significantly by mimics of miR-494 *in vitro* compared with control cell. (**B**,**C**) Moreover, the change of migration ability induced by mimics of miR-494 in mesenchymal phenotypic cell lines SNU-449, SK-HEP1, and PLHC-1 was more than that of basal epithelial phenotype cell lines PLC/PRF/5, Hep3B, and HepG2, while the difference between the two type panel of cell lines was statistically significant (*P* < 0.01). (**B**,**C**) Conversely, the abilities of migration in hepatoma cell lines were suppressed by inhibitors of miR-494. (**B**,**C**) Furthermore, the change of migration ability induced by inhibitors of miR-494 in mesenchymal phenotypic cell lines SNU-449, SK-HEP1, and PLHC-1 was less than that of basal epithelial phenotype cell lines PLC/PRF/5, Hep3B, and HepG2, while the difference between the two type panel of cell lines was statistically significant (*P* < 0.01). (**B**,**C**) The data are presented as means ± SD from three independent experiments. *P < 0.05, **P < 0.01. The mesenchymal markers expression of α-SMA, SMAD 3 and p-SMAD 3 in hepatoma cell lines were induced significantly by mimics of miR-494 *in vitro* compared with control cell. But, the expression of mesenchymal cell markers in hepatoma cell lines were suppressed by inhibitors of miR-494 in hepatoma cell lines (**D**).

To assess the biofunctions of miR-494, over-expressed and down-expressed miR-494 was conducted, respectively. Furthermore, several molecular functional tests were performed in hepatoma cell lines. The proliferation ability of hepatoma cell lines was enhanced by miR-494 compared to that of control (Fig. 6A). Moreover, the change of proliferation ability induced by mimics of miR-494 in mesenchymal phenotypic cell lines SNU-449, SK-HEP1, and PLHC-1 was significantly more than that of basal epithelial phenotype cell lines PLC/PRF/5, Hep3B, and HepG2, but the difference was not statistically significant (*P* > 0.05) (Fig. 6A).

Additionally, the inhibitors of miR-494 suppressed proliferation ability of hepatoma cell lines (Fig. 6A). Furthermore, the change of proliferation ability induced by inhibitors of miR-494 in mesenchymal phenotypic cell lines SNU-449, SK-HEP1, and PLHC-1 was less than that of basal epithelial phenotype cell lines PLC/PRF/5, Hep3B, and HepG2, while the difference between the two type panel of cell lines was statistically significant (*P* < 0.01) (Fig. 6A). It identified that miR-494 promoted the process of proliferation in hepatoma cell lines, especially for mesenchymal phenotypic cell lines.

The migration ability of hepatoma cell lines was enhanced significantly by miR-494 mimics *in vitro* compared with control group (Fig. 6B,C). Moreover, the change of migration ability induced by mimics of miR-494 in mesenchymal phenotypic cell lines SNU-449, SK-HEP1, and PLHC-1 was more than that of basal epithelial phenotype cell lines PLC/PRF/5, Hep3B, and HepG2, while the difference between the two type panel of cell lines was statistically significant (*P* < 0.01) (Fig. 6B,C).

Conversely, the abilities of migration in hepatoma cell lines were suppressed by inhibitors of miR-494 (Fig. 6B,C). Furthermore, the change of migration ability induced by inhibitors of miR-494 in mesenchymal phenotypic cell lines SNU-449, SK-HEP1, and PLHC-1 was less than that of basal epithelial phenotype cell lines PLC/PRF/5, Hep3B, and HepG2, while the difference between the two type panel of cell lines was statistically significant (*P* < 0.01) (Fig. 6B,C). Our results identified miR-494 enhanced migration ability of hepatoma cell lines.

### MiR-494 induced mesenchymal markers expression of hepatoma cell lines

The mesenchymal markers expression of α-SMA, SMAD 3 and p-SMAD 3 in hepatoma cell lines were induced significantly by mimics of miR-494 *in vitro* compared with control group (Fig. 6D). But, the expression of mesenchymal cell markers in hepatoma cell lines were suppressed by inhibitors of miR-494 in hepatoma cell lines (Fig. 6D). Our results identified miR-494 promoted mesenchymal markers expression of α-SMA, SMAD 3 and p-SMAD 3 in hepatoma cell lines.

### MiR-494 antagomir suppressed HCC xenografts

**Figure 7:**
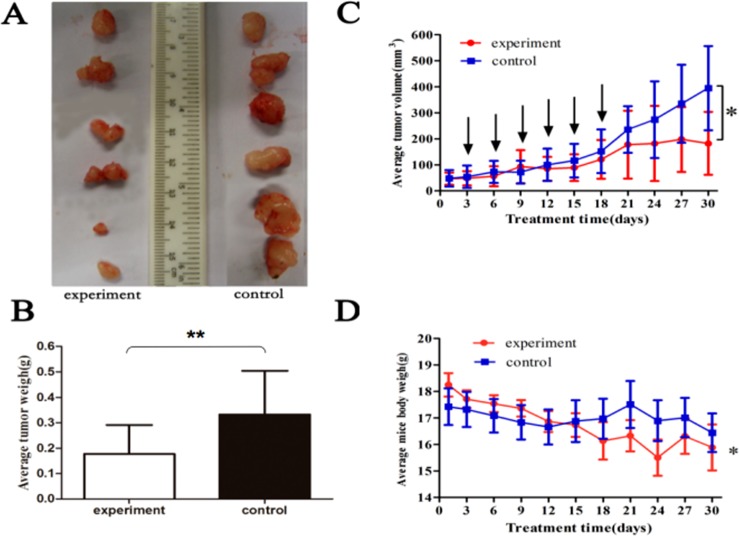
MiR-494 antagomir suppressed development of HCC xenografts. To detect the anti-tumor activity of chaetocin *in vivo*, human HCC xenografts were established with HepG2 cells. The results showed that the xenografts of control groups grew rapidly, but in experiment group, which was treated with antagomir of miR-494, grew slowly *in vivo* (**A–C**), moreover, the weight of tumors in experiment group (treated with antagomir of miR-494) was significantly less than that of control group. (**B**) At the time of end, the average tumor volume of control group was much larger than that of experiment group, and the difference between two groups had statistical significance. (**D**) The data are presented as means ± SD from three independent experiments. *P < 0.05, **P < 0.01.

**Figure 8:**
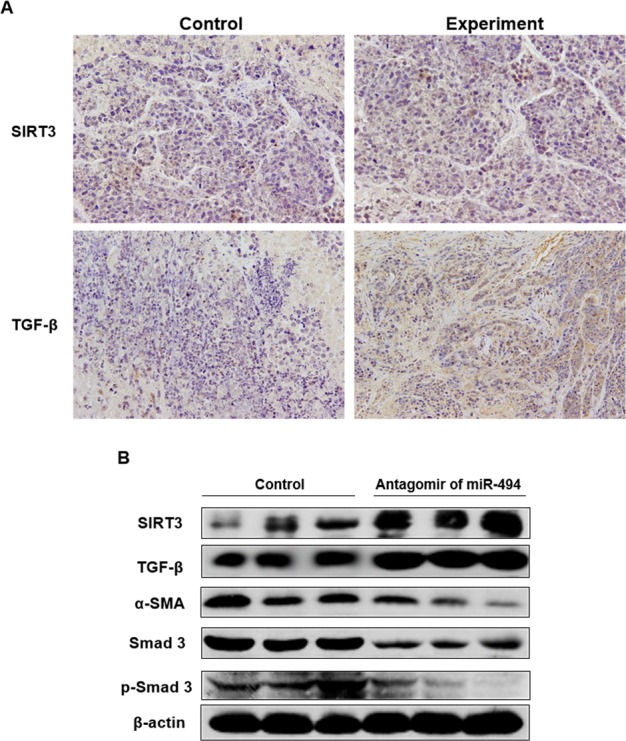
The miR-494 antagomir suppressed expression of mesenchymal cell markers in xenografts. The expression proteins in xenografts was determined with immunohistochemical staining (**A**) and western blotting assay. (**B**) Compare with control group, treatment with antagomir of miR-494 could up-regulate the expression of SIRT3 and TGF-β with a statistically significant, and suppressed mesenchymal markers expression of xenograft. The mesenchymal markers expression of α-SMA, SMAD 3 and p-SMAD 3 in xenografts were performed. The results indicated that compare with control group, treatment with antagomir of miR-494 could obviously down-regulate the expression of the above mesenchymal cell markers in xenografts.

To detect anti-tumor activity induced by miR-494 antagomir *in vivo*, human HCC xenografts were established with HepG2 cells. Our work demonstrated xenografts of control groups grew rapidly, but in the experiment group, which was treated with antagomir of miR-494, grew slowly *in vivo* (Fig. 7A–C). Moreover, the weight of tumors in the experiment mice (treated with antagomir of miR-494) was significantly less than that of control group (Fig. 7B). At the time of end, the average tumor volume of control mice was much larger than that of experiment group, and the difference between two groups had statistical significance (Fig. 7D). Therefore, the antagomir of miR-494 could obviously inhibit tumor proliferation and development process. In addition, the average weighed of mice heavier in early stage of the experiment was less than that in late stage and with a statistical significance, but it was not observed in the control group. The expression proteins in xenografts were determined with immunohistochemical staining (Fig. 8A) and western blotting methods (Fig. 8B), respectively. As we can see, the results revealed that when compare with control group, treatment with antagomir of miR-494 could up-regulate the expression of SIRT3 and TGF-β with a statistically significant, and suppressed mesenchymal markers expression of xenograft. The mesenchymal markers expression of α-SMA, SMAD 3 and p-SMAD 3 in xenografts were determined. The results indicated that compare with control group, treatment with antagomir of miR-494 could obviously down-regulate the above mesenchymal markers expression in xenografts. To sum up, our data confirmed that the antagomir of miR-494 could inhibit development of HCC associated with EndMT throught aiming SIRT3/TGF-β signaling pathway *in vivo*.

## Discussion

Through EndMT (endothelial-mesenchymal transition), a mesenchymal phenotype in endothelial cells can be acquire, wherein the marker expression and acquired functions of mesenchymal cell, in addition to lost the endothelial functions and markers expression of endothelia. Besides, migration and delamination of endothelial cells can be caused by EndMT, then mesenchymal cells derived from endothelial cell can migrate into the underlying tissue^39^. The ligands of TGF-β (transforming growth factor β) family often initiate the cellular process, EndMT^40^. Many investigations aim at the markers of EndMT from thirty-two cancer types^41,42^ and the roles of TGF-β pathway^41,43^. A number of elements are important factor in regulation of gene expression and EndMT, which referred to development of HCC^44–47^.

Moreover, the transcriptional regulation mechanism of EndMT and how miRNA modulated EndMT and development of HCC was unclear yet. Recent research revealed that through down-regulating directly SIRT1 and c-Myc loop, miR-494 acts as inhibitor and predictor of pancreatic cancer^48^. Moreover, through suppressing FGFR2, the lapatinib resistance can be reversed and the phenotype of cancer-initiating cell can be inhibited by miR-494 in HER2-positive gastric cancer^49^. But, on the contrary, Sun *et al*. confirmed miR-494 over-expression in colorectal cancer was related to metastasis and aggressiveness of tumor, while enhanced invasion and migration through aiming PTEN. Besides, for patients with colorectal cancer, miR-494 acted as the independent marker for prognostic^50^. Mao *et al*. demonstrated that through HIF-1α mediated mechanism, hypoxia in tumor cells induced miR-494 expression. Moreover, the specific antagomiR of miR-494 could suppress efficiently tumor growth xenografts and attenuate angiogenesis of nude mice^51^. Therefore, in this study, miR-494 was ascertained and searched after its effects on EndMT and development process of HCC.

At first, the differential miRNAs expression profiles between tumorous and para-tumorous tissues were assessed with miRNA-sequencing experiments. Then, we found that, compared with para-tumorous tissues, 17 miRNAs were upregulated (included miR-494) and 3 miRNAs were down-regulated in tumorous tissues of patients with HCC. Furthermore, the relative expression level of miR-494 in non-tumorous, para-tumorous, and tumor tissues were determined using qRT-PCR method. This work identified relative levels of miR-494 expression in tumorous tissues were obviously much more than that in para-tumorous and non-tumorous tissues. However, there was no obvious difference between para-tumorous and non-tumorous tissues. Consequently, the relative miR-494 expression levels were investigated in six cell lines of human HCC. The result showed that miR-494 over-expression was related to phenotype of EndMT, compared with basal epithelial phenotype cell lines PLC/PRF/5, Hep3B, and HepG2, the relative miR-494 expression levels were significantly higher in mesenchymal phenotypic cell lines SNU-449, SK-HEP1, and PLHC-1.

Therefore, the targets of miR-494 were foretasted with Targetscan, an online software. Therefore, SIRT3 was found that possibly aimed to 3′UTRs of SIRT3 mRNA. Then, further experiments were performed and identified that miR-494 could straight bind with 3′UTRs of SIRT3 mRNA. In a word, miR-494 directly argeted to SIRT3 mRNAs. It indicated that miR-494 could straight control mRNA and the protein expression levels of SIRT3, further affect expression level of TGF-β protein in hepatoma cell lines. Taken together, our study maybe point out a novel aim at treatment for HCC associated with EndMT.

Furthermore, immunohistochemistry assay was conducted on liver tissues of patients, too. The results demonstrated that SIRT3 was positive in all cases. However, the positive intensity and express location in different groups were differences. SIRT3 mainly appeared in the cytoplasm in tumor tissue, but they were at the same time appearing in the cytoplasm and nucleus of para-tumorous and non-tumorous tissues. Moreover, the positive intensity of tumor tissue much lower than para-tumorous or non-tumorous tissues. The differences of SIRT3 positive intensity between tumorous and para-tumorous or non-tumorous tissue were statistically significant. In summary, it indicated the definite relationship between the expression level of SIRT3 protein and its contribution to the occurrence of HCC.

Besides, to assess the biofunctions of miR-494, several molecular functional tests were performed in hepatoma cell lines transfected with mimics or inhibitors of miR-494, respectively. It identified miR-494 enhanced proliferation process and the migration ability of hepatoma cell lines, while significantly promoted mesenchymal markers expression of α-SMA, SMAD 3 and p-SMAD 3 in hepatoma cell lines.

Furthermore, the effects on Human HCC xenograft and its mechanism were investigated using miR-494 antagomir. Our results comfirmed that the xenografts of control groups grew rapidly, but in the experiment group, which was treated with antagomir of miR-494, grew slowly *in vivo*. Moreover, the weight of tumors in experiment group (treated with antagomir of miR-494) was significantly less than that of control group. At the time of end, the average tumor volume of control group was much larger than that of experiment group, and the difference between two groups had statistical significance. In addition, the average weighed of mice heavier in early stage of the experiment was less than that in late stage and with a statistical significance, but it was not observed in the control group. Therefore, the antagomir of miR-494 could obviously inhibit tumor proliferation and its development process. Moreover, treatment with antagomir of miR-494 could up-regulate the expression of SIRT3 and TGF-β with a statistically significant, and suppressed the expression of mesenchymal cell markers in xenograft.

We thought that our manuscript obviously was composed of only the whole one part. The assays *in vivo* and *in vitro* were all separately used to identify our conclusions. In summary, we illustrated that miR-494 targets to SIRT3. Moreover, miR-494 was a crucial mediator of EndMT and the development of HCC through regulating SIRT3/TGF-β/SMAD signaling pathway. It suggested that aim at SIRT3/TGF-β/SMAD signaling pathway through restraining miR-494 expression, was a feasible therapy strategy for HCC.

## Supplementary information

Supplementary informations
