# Supplementary material for: miR-494 induces EndMT and promotes the development of HCC (Hepatocellular Carcinoma) by targeting SIRT3/TGF-β/SMAD signaling pathway
Source: Sci Rep. 2019 May 10;9:7213. doi: 10.1038/s41598-019-43731-4 (PMC6510769; doi:10.1038/s41598-019-43731-4)
Supplement: Supplementary file 1 — Supplementary informations [file 41598_2019_43731_MOESM1_ESM.doc]

**miR-494 induces EndMT and promotes the development of HCC (Hepatocellular Carcinoma) by targeting SIRT3/TGF-**β**/SMAD signaling pathway**

Jinqian Zhang^1*^, Yan Zhu^1^, Liangshan Hu^2^, Fang Yan^2^, Jinglong Chen^3*^

1. Department of Laboratory Medicine and Central Laboratories, Guangdong Second Provincial General Hospital, Guangzhou 510317, PR China
2. Department of Medical Experiment Center, Guangdong Second Provincial General Hospital, No.466 Xingang Middle Road, Haizhu District, Guangzhou 510317, Guangdong Province, China
3. Department of Oncology, Beijing Ditan Hospital, Capital Medical University, Beijing 100015, PR China

***Corresponding author:** Prof. Jinqian Zhang, Department of Laboratory Medicine and Central Laboratories, Guangdong Second Provincial General Hospital, Guangzhou 510317, PR China, or Email: [jingwanghou@163.com](mailto:jingwanghou@163.com); and Prof. Jinglong Chen, Department of Oncology, Beijing Ditan Hospital, Capital Medical University, Beijing 100015, PR China, or Email: [dtzlzx@sina.com](mailto:jianshengliylp@163.com)

**Short title:** miR-494 induces EndMT and promotes HCC by targeting SIRT3/TGF-β/SMAD signaling pathway

**1. Data extraction and analysis**

Data extraction and analysis of miRNAs next-genersequencing was performed as showed in Flow diagram (**Supplement Figure 1**).


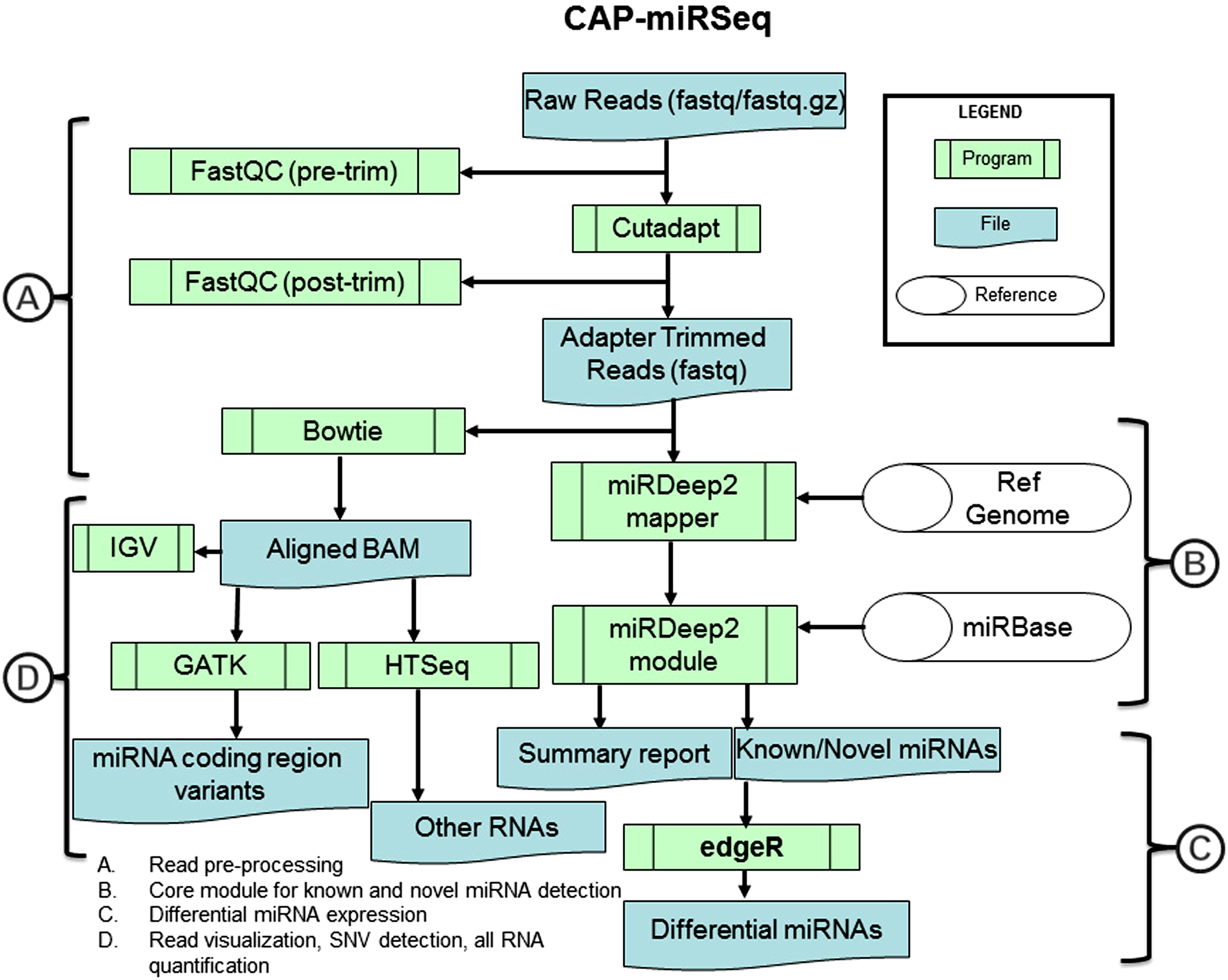


**Supplement Figure 1.** Data extraction and analysis of next-genersequencing miRNAs was performed as showed in Flow diagram.

1. **Predicted consequential pairing of target region.**

The online software Targetscan (version 7.2) was firstly used to forecast the microRNA which could regulate the expression of SIRT3. Therefore, miR-494 was acquired from predict result. The complementary region sequence in SIRT3 3′UTR was UUUC(AAGAUG)AUGUUUC (Supplementary Table S1), which located at position 1618-1624, and corresponding miR-494 seed sequence CUCCAAAGGGCACAUACAAAGU.

|  | **Predicted consequential pairing of target region (top) and miRNA (bottom)** | **Site type** | **Context++ score** | **Context++ score percentile** | **Weighted context++ score** | **Conserved branch length** | **P_CT_** |
| --- | --- | --- | --- | --- | --- | --- | --- |
| Position 1618-1624 of SIRT3 3' UTR  [hsa-miR-494-3p](http://www.mirbase.org/cgi-bin/mirna_entry.pl?acc=hsa-miR-494-3p) | 5' ...CUUUAUUUCAAGAUGAUGUUUCU...            ||||      |||||||  3'     CUCCAAAGGGCACAUACAAAGU | 7mer-m8 | -0.05 | 76 | 0.00 | 2.862 | N/A |

**Supplement Table 1. Predicted consequential pairing of target region.**
